# Rapid pre-retinal ossification presenting as a vascularized lesion over an area of chronic retinal detachment

**DOI:** 10.1016/j.ajoc.2022.101791

**Published:** 2022-12-31

**Authors:** Chu Jian Ma, Melike Pekmezci, Jay M. Stewart

**Affiliations:** a University of California San Francisco, Department of Ophthalmology, San Francisco, CA, USA; b Zuckerberg San Francisco General Hospital and Trauma Center, Department of Ophthalmology, San Francisco, CA, USA; c University of California San Francisco, Department of Pathology, San Francisco, CA, USA

**Keywords:** Osseous metaplasia, Pre-retinal fibrosis, Chronic retinal detachment, Recurrent retinal detachment

## Abstract

**Purpose:**

To describe the clinical, optical coherence tomography (OCT), and histopathological findings of a patient who was found to have ossification of a pre-retinal membrane after multiple surgical repairs for retinal detachment.

**Methods:**

The patient had comprehensive ophthalmic examinations during seven years of follow-up and underwent surgical removal of her pre-retinal membrane.

**Results:**

A 24-year-old woman with a history of retinal detachment and multiple retina surgeries presented with baseline vision of 20/200 and refractory glaucoma in the left eye (right eye with no light perception due to prior failed retinal detachment repair). OCT showed a thick epiretinal membrane with hypo-reflective intraretinal spaces in the macula, and exam revealed a chronic retinal detachment superotemporally surrounded by laser barricade. She was stable for six years and then experienced vision loss and decreasing eye pressure, concurrent with rapid evolution of pre-retinal fibrosis, leading to a vascularized consolidation in the mid-periphery, for which she underwent vitrectomy and membrane peel. The vascularized lesion over the area of detachment in the superotemporal retina was removed en bloc through the anterior chamber. Pathological findings revealed woven bone formation anterior to the internal limiting membrane, and the tissue was GFAP negative.

**Conclusions:**

Our case adds to the limited knowledge of the chronology, presentation, and surgical management of intraocular ossification, especially of the rarer pre-retinal type. Our patient highlights that development of ossification can happen more quickly than previously thought (year or years rather than decades), can be hidden under vascularized lesions, and is dynamic, with simultaneous release of traction in one area and increased traction in another. Diligent follow-up is indicated even in cases of vitreous membranes from retinal detachment that otherwise appear to have been stable for years.

## 1 Introduction

Intraocular osseous metaplasia is a rare occurrence, described usually in phthisical eyes or eyes with poor vision (hand motions or worse) secondary to chronic retinal detachment, trauma, or uveitis years after onset of disease.^1^, ^2^, ^3^ Given pain and poor vision, these eyes have usually eventuated to evisceration.^4^, ^5^, ^6^ Ossification was mostly discovered in enucleated eyes and often appeared sub- or intra-retinal, as it is thought to arise from the pluripotency of the retinal pigment epithelium.^7^^,^^8^ Since those studies were done with enucleated eyes, there is little known about the chronology of events leading to ossification, their impact on vision, or their management.

Here we report a case of dystrophic ossification in a focal fibrovascular lesion in the setting of a chronic retinal detachment after multiple surgical repairs followed over six years with rapid evolution of the lesion over several years, concurrent with drop in intraocular pressure in the absence of ocular inflammation. The patient's lesion was surrounded by a vascular capsule and localized in the pre-retinal space over an area of chronic retinal detachment, and bone formation and contraction were associated with concurrent release of traction over the macula. We also highlight the surgical technique of its removal, ultimately leading to improvement in the patient's vision.

## 2 Case report

**Fig. 1:**
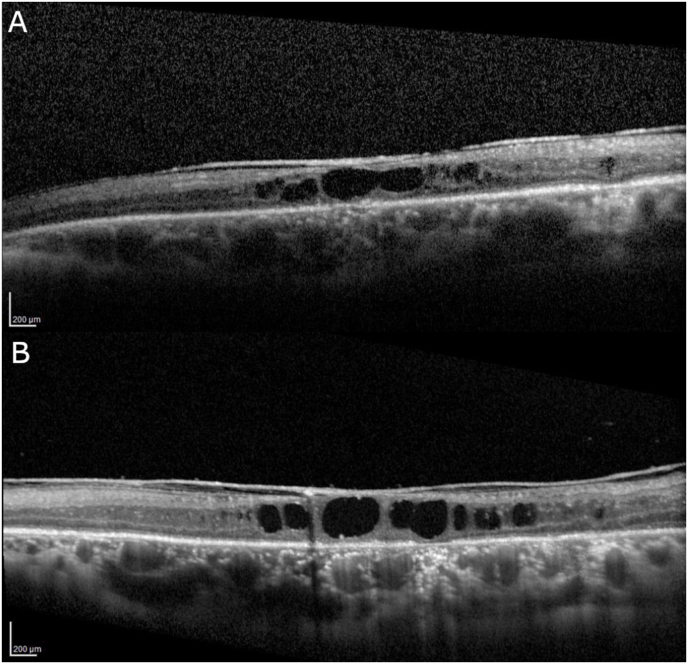
OCT (horizontal scan across fovea) showing retinal thinning in macula; large areas of hyporeflectivity consistent with intraretinal tissue loss, likely from prior chronic cystoid macular edema, and epiretinal membrane vs. posterior hyaloid membrane. (A) Year 1. (B) Similar findings in year 5.

**Fig. 2:**
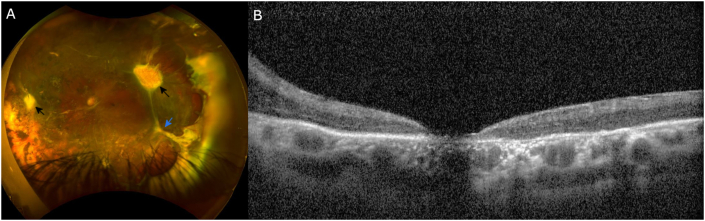
Fundus photo, and OCT (horizontal scan across fovea) of left eye (year 7). (A) Attached retina posterior to laser barrier; vitreous membrane with tractional connection to fibrovascular lesion superotemporally and nasally (black arrows), apparent traction to anterior loop fibrosis as well (blue arrow). (B) Compact macula with retinal thinning and outer retinal loss at fovea, resolution of previously seen intraretinal hyporeflective spaces in macula, concurrent with release of the ERM over the fovea. (For interpretation of the references to colour in this figure legend, the reader is referred to the Web version of this article.)

**Fig. 3:**
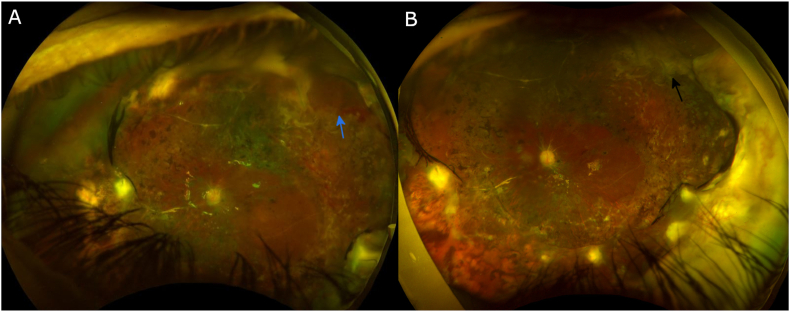
Postoperative fundus photo of left eye. (A) Post-op week 1: Attached retina posterior to laser barrier, minimal blood visible superotemporally (blue arrow). (B) Post-op month 16: Clear view to fundus with attached retina posterior to laser scars, remodeled fibrovascular membrane in far periphery (black arrow), no hemorrhages seen. (For interpretation of the references to colour in this figure legend, the reader is referred to the Web version of this article.)

A 24-year-old woman with high myopia, a history of bilateral retinal detachment status post multiple retinal surgeries, and secondary glaucoma presented to our retina clinic as an outside referral. Her medical history was notable for mild intermittent asthma and iron deficiency anemia, and she denied any significant family history of eye disease. Prior to her initial presentation to our clinic, she had already undergone one retinal detachment repair in the right eye (with scleral buckle) eight years earlier and four surgeries in the left eye (including one scleral buckle procedure and revision), with the last surgery done five years earlier, and she was aphakic in both eyes. The original cause of the detachments was not clear given the lack of clinical notes and surgical reports from initial operations done elsewhere; however, there was no documented history of trauma to the eyes. On presentation, her vision was no light perception in the right eye and 20/200 in the left eye, stable from one year ago comparing to outside records. Ophthalmic examination of the right eye revealed band keratopathy with poor views beyond the cornea, and B-scan ultrasonography showed retained silicone oil. The left eye's examination revealed aphakia, absence of vitreous following prior vitrectomy, pale and atrophic disc (0.9 cup-to-disc ratio), attached macula with 360° of laser scars beyond the vascular arcades, and multiple retinal holes, membranes and retinal detachment anterior to the laser, as well as an encircling scleral buckle. Optical coherence tomography (OCT) of the left eye showed retinal thinning in the macula with schisis-like cavities (large areas of hypo-reflectivity consistent with intra-retinal tissue loss along with traction from overlying epiretinal membrane) (Fig. 1). In year one, she underwent *trans*-scleral cyclophotocoagulation in the left eye for glaucoma management with reduction of her intraocular pressure (IOP) to the low to mid-teens. Her ophthalmic examination (without intraocular inflammation) and visual acuity in the left eye remained stable until year five. She was then lost to follow up for two years due to the COVID-19 pandemic, and in year seven, her visual acuity declined to 20/400 in the left eye, and IOP dropped to 7 mm Hg. At this point, examination of the left eye showed an attached retina posterior to the laser scars, a vitreous membrane with tractional connection to a fibrovascular lesion superotemporally and a lesion nasally, and traction to anterior loop fibrosis as well (Fig. 2A). OCT showed stable retinal thinning and outer retinal loss at the fovea and resolution of the previously seen hyporeflective spaces in the macula, along with separation of the epiretinal membrane (Fig. 2B). Two months later, the patient came in for an acute visit complaining of progressively smaller visual field over the previous weeks. The retina remained attached posteriorly, but given the symptoms and concern for possible progressive traction from the prominent insertions of the fibrovascular lesion superotemporally causing hypotony and vitreous hemorrhage, the patient was taken to surgery for membrane peel, endolaser, silicone oil placement, and off-label intravitreal methotrexate injection. During the surgery, the vitreous cutter was used to remove the pre-retinal membrane peripheral to the vascularized peripheral lesion. Cautery was then applied to the lesion, but it was too thick to be cauterized; therefore, endodiathermy was used to mark the sheets of fibrosis to detach the retina surrounding the lesion. The lesion was then removed from its connections using forceps but was too large and dense to be removed with the vitreous cutter, so it was brought to the anterior chamber with 23g forceps and removed from the eye through a limbal incision at 6 o'clock (where the pupil was most dilated). The specimen was sent for pathological analysis. Residual tractional membranes in the superotemporal quadrant were removed. The retina was flattened under air. The eye was filled with silicone oil, and intravitreal methotrexate (400 mcg) was administered. Postoperatively, the patient received intravitreal injections of methotrexate every two weeks for a total of five injections starting at post-op week one. The decision was made to keep the silicone oil in the eye over a longer term than usual, as the patient reported subjectively better vision with it in place. The final visual acuity at post-op month sixteen was 20/300 (stable to initial presentation), the exam showed that retina remained attached, and there was a remodeled fibrovascular frond at the edge of the prior resection (Fig. 3), and the OCT showed no macular edema with stable atrophy.

## 3 Pathological findings

**Fig. 4:**
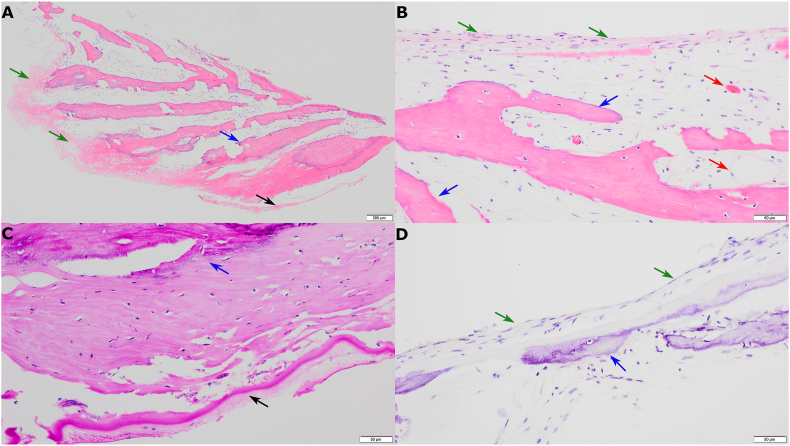
Pathologic features of the pre-retinal ossified lesion. (A) Hematoxylin and eosin staining, 40x magnification (ruler 200 μm), showing woven bone (blue arrow) in front of the internal limiting membrane (black arrow) and fibrous tissue (green arrows). (B) Hematoxylin and eosin staining, 200x magnification (ruler 50 μm), showing woven bone (blue arrow) adjacent to fibrous tissue (green arrows) with vasculature (red arrows). (C) Periodic acid-Schiff (PAS)-staining, 200x magnification (ruler 50 μm), highlighting the internal limiting membrane (black arrow) and the woven bone (blue arrow). (D) Fibrous tissue (green arrow) adjacent to ossified plaque (blue arrow) is negative for GFAP, 200x magnification (ruler 50 μm). (For interpretation of the references to colour in this figure legend, the reader is referred to the Web version of this article.)

The surgical specimen showed pre-retinal membrane, predominantly composed of woven bone formation (ossification) in front of the internal limiting membrane (Periodic acid-Schiff positive) (Fig. 4). No pigmented cells were seen to suggest retinal pigment epithelium (RPE). The tissue was GFAP-negative (glial fibrillary acidic protein), arguing against any significant gliosis or the presence of any retinal tissue deeper than the internal limiting membrane. Overall, the features are those of a predominantly pre-retinal membrane with dystrophic ossification.

## 4 Discussion

Prior reports of intraocular ossification have been postulated that it results from chronic inflammation and disorganization of the globe, often associated with phthisis after trauma or chronic unrepaired retinal detachment, or uveitis, with ossification as an end stage of disease evolution.^1^ Histological studies have demonstrated central calcium or bone formation surrounded by RPE cells, suggesting that RPE cells are the potential source for the cells which had undergone metaplastic changes.^6^^,^^9^^,^^10^ While the preretinal site of the osseous metaplasia is not adjacent to the normal location of RPE, it is possible that RPE cells might have been displaced to a preretinal location during multiple prior retinal surgeries. Osseous metaplasia is not unique to the eye and can be seen in many other organs and tissue types. Therefore, it is also conceivable that one of the prior surgeries could have brought in epithelium, fibroblasts or other cell types from extraocular sources to the vitreous cavity, although this is something that can neither be refuted nor proven based on the available histopathologic findings.

In many of the examples in the literature, pre-, intra- or subretinal ossification is associated with chronic retinal detachment, as it is seen in our case.^6^^,^^7^^,^^9^^,^^10^ Gliosis frequently accompanies the proliferative vitreoretinopathy, preretinal membranes as well as scar tissues within the retina. Accordingly, previous reports of intraocular ossification demonstrated GFAP-positive gliosis surrounding the ossified plaques.^3^^,^^7^ Interestingly, in our case the woven bone was immediately associated with internal limiting membrane and did not demonstrate associated GFAP-positive gliosis. We also did not see pigmented cells to suggest RPE. It is possible that the gliosis and RPE may have been entirely replaced by the advanced osseous metaplasia by the time the tissue was removed or that gliosis and RPE were only confined to an area further outside of the internal limiting membrane which was not sampled.

It is also interesting that although literature suggests that bone tissue in the eye requires decades to develop,^4^^,^^5^ our patient was followed over the span of eight years, and significant changes in the appearance of the membrane were only noticed in the final year. It is unclear whether the presence of silicone oil accelerated ossification, as one report suggested,^4^ or whether it was simply a bystander, as our patient only had a trace amount of silicone oil in the eye prior to her latest surgery. This also suggests that not all intraocular ossification processes are the same, and pre-retinal ossification might be different and occur faster than intra- or sub-retinal ones, likely due to the different extracellular environment once it is no longer surrounded by retinal tissue.^7^

As our patient was already unicameral (aphakic), we were able to remove the calcified lesion through the anterior chamber without the need for an additional sclerotomy. Using endodiathermy to relieve the traction from the fibrous membrane before removal of the calcified plaque also prevented intra-operative breaks that can occur with these procedures.^7^

In our patient, bone formation happened concurrently with contraction of the fibrovascular tissue and release of the epiretinal membrane traction upon the macula, leading to resolution of cystoid macular edema. Although calcification is not typically thought of as a dynamic process, our case highlights that it is at least associated with changes in the tractional forces on the retina and therefore should be monitored closely. In addition, ossification may not take as long to develop as had been thought and may warrant diligence in following even stable patients with chronic retinal detachments.

## Patient consent

Consent to publish the case report was not obtained. This report does not contain any personal information that could lead to the identification of the patient.

## Funding

Financial support: 10.13039/100001818Research to Prevent Blindness and All May See Foundation.

## Authorship

All authors attest that they meet the current ICMJE criteria for Authorship.

## Declaration of competing interest

The following authors have no financial disclosures: CJM, MP, JMS.
